# Membraneless organelles restructured and built by pandemic viruses: HIV-1 and SARS-CoV-2

**DOI:** 10.1093/jmcb/mjab020

**Published:** 2021-03-24

**Authors:** Viviana Scoca, Francesca Di Nunzio

**Affiliations:** 1 Advanced Molecular Virology and Retroviral Dynamics Group, Department of Virology, Pasteur Institute, Paris, France; 2 BioSPC Doctoral School, Universitè de Paris, Paris, France

**Keywords:** LLPS, MLO, HIV-1, SARS-CoV-2

## Abstract

Viruses hijack host functions to invade their target cells and spread to new cells. Specifically, viruses learned to usurp liquid‒liquid phase separation (LLPS), a newly exploited mechanism, used by the cell to concentrate enzymes to accelerate and confine a wide variety of cellular processes. LLPS gives rise to actual membraneless organelles (MLOs), which do not only increase reaction rates but also act as a filter to select molecules to be retained or to be excluded from the liquid droplet. This is exactly what seems to happen with the condensation of SARS-CoV-2 nucleocapsid protein to favor the packaging of intact viral genomes, excluding viral subgenomic or host cellular RNAs. Another older pandemic virus, HIV-1, also takes advantage of LLPS in the host cell during the viral cycle. Recent discoveries highlighted that HIV-1 RNA genome condensates in nuclear MLOs accompanied by specific host and viral proteins, breaking the dogma of retroviruses that limited viral synthesis exclusively to the cytoplasmic compartment. Intriguing fundamental properties of viral/host LLPS remain still unclear. Future studies will contribute to deeply understanding the role of pathogen-induced MLOs in the epidemic invasion of pandemic viruses.

## Introduction

Eukaryotic cells evolved mechanisms to ensure the performance of complex cellular functions in a limited space and in a spatiotemporal manner. Macromolecule condensates in cells, typically termed liquid-like droplets, are generated by liquid‒liquid phase separation (LLPS). LLPS is based on multivalent interactions among intrinsically disordered regions (IDRs) and/or modular interacting domains of some components (Bergeron-Sandoval et al., 2016; Banani et al., 2017; Shin and Brangwynne, 2017). IDRs do not have a well-defined structural conformation and usually contain repeated low-complexity sequences that favor transient intermolecular interactions such as aromatic, polar, and charge–charge (Bergeron-Sandoval et al., 2016; Banani et al., 2017; Shin and Brangwynne, 2017). As an example, IDRs characterize many RNA-binding proteins involved in the formation of large ribonucleoprotein (RNP) complexes. These proteins can form membraneless organelles (MLOs) using different RNA forms, including messenger RNA (mRNA), ribosomal RNA (rRNA), long noncoding RNA (lncRNA), small nuclear RNA, and small nucleolar RNA, as molecular scaffold for their condensation (Shin and Brangwynne, 2017; Fay and Anderson, 2018). Thus, MLOs are typically formed via LLPS, generated by an equilibrium between particular molecules concentrated in a liquid-like compartment and the surrounding liquid milieu (Banani et al., 2017; Shin and Brangwynne, 2017). This cellular organization allows a range of distinct cellular functions in a confined space (Boeynaems et al., 2018). MLOs have aroused the interest of many scientists from different disciplines, because they are critical for many biological phenomena. Some studies suggest an old origin of MLOs; indeed, they can be composed of simple heterogeneous polymer systems similarly to synthetic products from early Earth (Yoshizawa et al., 2020).

MLOs include biomolecular condensates, such as processing bodies, stress granules (SGs), nuclear pore complexes (NPCs), paraspeckles, speckles, promyelocytic leukemia nuclear bodies (PML NBs), the nucleolus, DNA damage foci, transcription factories, and germline granules. It has been observed that the composition of MLOs can be responsible for their distinct biological functions and their dynamic state (Alberti and Hyman, 2016; Kaganovich, 2017). In fact, condensates can play divergent biological roles (Roden and Gladfelter, 2020): (i) triggering the interaction between factors by bringing them closer to each other (Seydoux and Braun, 2006; Lee et al., 2013, 2015; Wang and Seydoux, 2014; Langdon and Gladfelter, 2018), (ii) serving as sink for chemical reactions (Lu et al., 2018), (iii) enhancing enzymatic rates (Hnisz et al., 2017; Cho et al., 2018), and (iv) acting as stress sensors (Riback et al., 2017; Du and Chen, 2018). Phase separation plays also a role in cargo trafficking pathways, such as docking cargos to mediate their transport across membranes and shuttling cargos through the NPC. Importantly, membrane-bound and MLOs orchestrate actions to guarantee spatiotemporal control of multiple cellular functions. In fact, LLPS plays broad roles with membrane-associated structures, such as postsynaptic density (PSD) in neurons and T cell signaling. Phase separation-mediated PSD assembly can determine the physiological functions of synapses (Dustin and Choudhuri, 2016; Dustin and Kam, 2016; Zeng et al., 2016; Courtney et al., 2018). New evidences show that membrane-bound organelles and membraneless condensates closely interact, regulating various functions of both types of organelles that can be usurped by viruses for their replication (Zhao and Zhang, 2020). Study based on advanced electron microscopy revealed that positive-sense RNA viruses, such as picornaviruses, hepatitis C virus, noroviruses, and coronaviruses usurp host membranes to generate viral replication organelles, inducing encapsulated spherules or double-membrane vesicles (DMVs) for viral RNA (vRNA) synthesis (Knoops et al., 2008; Maier et al., 2013; Zhang et al., 2018; Snijder et al., 2020; Wolff et al., 2020). However, it is still unclear how critical DMVs are for effective viral spread, which is a vital notion for the design of broad-acting antivirals. Phase separation plays a key role in genome organization and gene expression; in fact, the nucleus optimizes its intricate function through its own compartmentalization. For instance, the genomic 3D organization is pivotal not only for the packaging of several Mb of DNA in the small nuclear volume but also for short- and long-range interactions between regulatory sequences and genes. Nuclear architecture is dynamically coordinated by DNA-interacting proteins, which cleverly cluster the chromatin for the best purpose. The assembly of a large amount of specific nuclear factors, which transiently or permanently interact with DNA and/or RNA, generate well-defined nuclear domains (nuclear bodies). Even though biomolecular condensates miss an actual barrier from the surrounding environment, they represent actual independent ‘factories’. Overall, it is extremely advantageous for the cell exploiting these structures to quickly respond to different environmental inputs and/or to cellular perturbations, such as viral invasion. Indeed, viruses evolved multiple mechanisms to adapt and coexist with the host to be able to release their new progeny. For example, DNA and RNA viruses learned to build or restructure MLOs to replicate.

This review focuses on the interplay between MLOs and two pandemic viruses: HIV-1 and SARS-CoV-2. HIV-1 virions carry two copies of RNA genome that should be retrotranscribed to allow viral integration into the host chromosomal DNA. Thus, HIV-1 replicates in the nucleus of the host cell. Contrary to HIV-1, SARS-CoV-2, which has a genome formed by single-stranded positive-sense RNA, replicates in the host cytoplasm (Zhou et al., 2020). This is also the case of SARS-CoV and SARS-related bat coronaviruses. Despite the differences in the cell cycle of those two categories of viruses, HIV-1 and SARS-CoV-2, recent studies highlight that both benefit from MLOs to replicate (Iserman et al., 2020; Perdikari et al., 2020; Rensen et al., 2020; Savastano et al., 2020; Scoca et al., 2020;  Cubuk et al., 2021). This review aims to discuss the most important strategies evolved by these two pandemic viruses to interact with MLO components for an efficient sabotage of the cellular compartments to their own benefit.

## HIV-1 and SARS-CoV-2 interplay with the host MLOs

### SGs

Phase separation dictates the principles of cell organization, governing cell function and survival (Uversky, 2017), for instance, generating SGs in response to environmental and stress factors (Franzmann and Alberti, 2019). Of note, SGs can be induced by cellular stress, specifically triggered by translational silencing, causing accumulation of cellular mRNA. In most of the cases, the block of translation is due to the phosphorylation of the translation eukaryotic initiation factor 2α (eIF2α) (Protter and Parker, 2016). Different eIF2α kinases can sense environmental stress, like protein kinase RNA-dependent (PKR) in response to viral double-stranded RNA sensing (Bou-Nader et al., 2019) and PKR-like endoplasmic reticulum (ER) kinase (PERK/PEK) in response to hypoxia and misfolded proteins in the ER (Harding et al., 2000).

Numerous viruses, however, inhibit SG assembly to evade the antiviral response (Poblete-Duran et al., 2016). An example is HIV-1 that evolved multiple mechanisms to block the assembly of SGs through the interplay between the viral structural precursor polyprotein (pr55Gag) and several host factors such as eEF2, G3BP1, CypA, and eIF4E (Cinti et al., 2016). Interestingly, it has been proposed that during HIV-1 replication, an equilibrium exists between SG assembly and disassembly (Rao et al., 2018). More recently, it has been identified that a prion-like IDR conserved among retrovirus Gag proteins regulates their zinc-dependent LLPS. This LLPS drives nucleocapsid-stress granule (NC-SG) formation and, in the presence of vRNA, viral RNP assembly. The chelation of the Zn^2+^ blocks the development of these MLOs and induces a relocalization of nucleocapsid and viral genomic RNA (NC-vRNA). The infection outcome seems to be based on the NC-vRNA/NC-SGs balance, which is due to the ordered Zn^2+^ LLPS of NC proteins that contributes to viral assembly, while the NC-SGs avoid an excessive accumulation, as NC proteins have a tendency to multimerize (Table 1; Monette et al., 2020).

**Table 1:** Roles of MLOs in viral infection of pandemic viruses HIV-1 and SARS-CoV-2.

MLOs	Viruses: HIV-1 and SARS CoV-2	Roles of MLOs during infection	References
SGs	HIV-1	Regulation of genomic RNA and trafficking	Rao et al. (2018); Monette et al. (2020)
	SARS-CoV-2	Role of N protein in SARS-CoV-2 viral genome packing	Iserman et al. (2020); Perdikari et al. (2020); Savastano et al. (2020); Cubuk et al. (2021)
NPC	HIV-1	Nuclear import and integration site selection	Di Nunzio et al. (2013); Lelek et al. (2015); Marini et al. (2015); Buffone et al. (2018)
	SARS-CoV-2	Inhibition of IFN signaling	Miorin et al. (2020)
Nucleolus	HIV-1	Viral RNP assembly platform	Michienzi et al. (2000)
	SARS-CoV-2	Untranslated viral genomic RNA accumulation	Wu et al. (2020)
PML NBs	HIV-1	Silenced HIV-1 location	Lusic et al. (2013)
NSs	HIV-1	Potential sites of the PIC maturation	Scoca et al. (2020)
		Nuclear reverse transcription	Burdick et al. (2020); Dharan et al. (2020); Rensen et al. (2020); Scoca et al. (2020); Selyutina et al. (2020)

Apart from HIV-1, several studies have been performed on the interplay of SARS-CoV with SGs. The infection, as for many other viruses, activates PKR and PERK kinases inducing SG assembly, but viral replication is not affected by eIF2α hyperphosphorylation, meaning that the viral expression is optimized in other ways, overcoming the induced cellular stress (Krahling et al., 2009). It has been observed that the host cell translation is hampered by mechanisms involving nonstructural protein 1 (nsp1) in SARS-CoV-infected cells (Kamitani et al., 2006, 2009; Narayanan et al., 2008; Lokugamage et al., 2012), which leads to stress-induced RNA accumulation (Anderson and Kedersha, 2008). *In vitro*, it has been observed that the nucleocapsid protein of SARS-CoV (SARS-CoV N protein) is recruited to SGs via its serine–arginine (SR) domain that can be phosphorylated at multiple sites by SRPK1 (Peng et al., 2008), the mammalian homolog of a yeast SR-kinase that regulates SGs (Shattuck et al., 2019). Of note, the protein N can interact with the granule-associated protein heterogeneous nuclear RNP A1 (hnRNPA1) (Luo et al., 2005) and other SG and phase-separating proteins (Molliex et al., 2015).

With COVID-19 outbreak, new research lines opened in the field of SARS-CoV-2 cellular stress. Similarly to the HIV-1 NC protein, the SARS-CoV-2 N protein undergoes phase separation with RNA *in vitro* (Perdikari et al., 2020). It has been hypothesized that this process facilitates SARS-CoV-2 replication by recruiting the excess of cytoplasmic proteins induced by the viral stress, like hnRNPs, as hubs to assemble dense N protein-viral genomic RNA phases to promote viral replication (Table 1; Perdikari et al., 2020). Importantly, proteomic studies performed on a putative SARS-CoV-2 protein interaction map identified some RNA processing factors and SG regulation factors, such as G3BP1/2, to be the epicenter of the N interactome (Gordon et al., 2020). In fact, G3BP1 and G3BP2, which drive the formation of SGs (Guillen-Boixet et al., 2020; Sanders et al., 2020; Yang et al., 2020), coprecipitate with the N protein (Gordon et al., 2020).

Taking into account studies on SARS-CoV, several models can be foreseen to explain the N protein role in SARS-CoV and SARS-CoV-2 life cycle: (i) N protein is passively recruited by SGs exercising no effect on them or it can actively play a role in turning down the host translation in favor of viral replication (Peng et al., 2008; Krahling et al., 2009); (ii) noncanonical SGs can be restructured and built during infection for viral replication or viral mRNA translation (Piotrowska et al., 2010; Scholte et al., 2015; Hou et al., 2017; Hosmillo et al., 2019; Brocard et al., 2020; Burke et al., 2020); and (iii) N protein may inhibit the formation of SGs via the sequestration of critical SG components, such as G3BP1, G3BP2, and hnRNPA1 (Cascarina and Ross, 2020). The relevance of the role of N protein for SARS-CoV-2 viral life cycle shed light on potential treatments that can be foreseen to target the interaction between N protein and host cell kinases or virus-induced MLOs to combat SARS-CoV-2 infection.

### NPC

The NPC regulates the exchange of components between the cytoplasm and the nucleus and represents the first barrier that viruses encounter to pass through the nuclear membrane. It is composed by ∼30 nucleoporins (Nups) (D'Angelo and Hetzer, 2008), which cover several functions, from the static scaffolding to the dynamic shuttling of cargos (Rabut et al., 2004) through the inner channel of ∼39 nm (Pante and Kann, 2002). One third of Nups contain phenylalanine‒glycine (FG) repeats (Raices and D'Angelo, 2012), which represent low-complexity IDRs. Most IDRs have a relatively low hydrophobicity and high net charge (Uversky et al., 2000), preventing the collapse into water insoluble aggregates, allowing the maintenance of their unfolded state in solution under physiological conditions (Monsellier and Chiti, 2007). A recent manuscript (Celetti et al., 2020) demonstrated that FG-Nups can undergo LLPS and form liquid droplets that mimic permeability barrier properties of intact NPCs. Thus, the authors evaluated whether the liquid Nup droplets have NPC-like permeability barrier properties, similarly to their solid counterparts. Liquid FG-Nup drops were rapidly penetrated by cargo–nuclear transport receptor (NTR) complexes, but only in the presence of the correct nuclear localization signal (NLS) and cognate NTR. Interestingly, this behavior was also observed for much larger cargo model, the recombinant capsid from MS2 bacteriophage; when surrounded by NLSs and importin α/β, this gigantic cargo was able to accumulate in FG-Nup drops (Celetti et al., 2020).

Nups are responsible for the static scaffolding of the NPC, as well as its dynamic transporter aspect. The dynamic and flexible nature of the NPC regulates the nuclear passage of viral complexes, such as for HIV-1 (Di Nunzio, 2013). The viral capsid is the determinant for HIV-1 nuclear import. Indeed, it has been demonstrated that the viral capsid directly docks at the NPC by engaging Nup358/RanBP2, located in the cytoplasmic side of the complex, by interacting with its Cyp-like domain (Di Nunzio et al., 2012). HIV-1 capsid also interacts with Nup153 (Table 1; Di Nunzio et al., 2013; Lelek et al., 2015), which is the most dynamic Nup, located in the nuclear side of the nuclear basket (D'Angelo and Hetzer, 2008). Interestingly, all Nups carrying FG domains can bind to HIV-1 capsid, probably helping with the efficiency of viral nuclear entry and, likely, post-nuclear entry steps (Buffone et al., 2018). Thus, HIV-1 capsid penetrates the liquid Nup droplets, likely formed by Nup153 (Celetti et al., 2020).

If on one side, HIV-1 exploits the NPC for its pre-integration complex (PIC) import in the nucleus, on the other, SARS-CoV-2 influences the functionality of the nuclear pore in a direct or indirect way. Nsp1 of SARS-CoV-2 is able to disrupt Nup93 localization around the nuclear envelope without triggering its proteolytic degradation or perturbation of the nuclear lamina. However, being the nuclear‒cytoplasmic exchange altered probably due to Nup93 impairment, a redistribution of the RNA binding protein nucleolin was observed (Gomez et al., 2019). Another recent study reported that ORF6 of SARS-CoV and SARS-CoV-2 inhibits STAT1 nuclear translocation to impede IFN signaling. Importantly, ORF6 localizes at the NPC where it directly interacts with the Nup98‒Rae1 complex to target the nuclear import pathway to overcome the antiviral action of IFN (Table 1; Miorin et al., 2020). Additional research might address more details on how SARS-CoV-2 can alter host nuclear import/export, through interaction with the NPC.

### Nucleolus

The nucleolus exemplifies the nucleolar MLO per excellence, which is further structured in three function-specific compartments, fibrillar centers (FCs), dense fibrillar component (DFC), and granular component (GC), all involved in different steps of rRNA biogenesis (Scheer and Hock, 1999). The nucleolar factors are condensed around the tandemly repeated ribosomal DNA (rDNA) and at the boundary between the FCs. In the DFC, there is a high concentration of Pol I for rDNA transcription; on the other hand, fibrillarin and small nucleolar RNPs (snoRNPs) are also enriched in the DFC, ready to process pre-rRNAs, which eventually are assembled in the GC (Boisvert et al., 2007). Nucleolar RNPs constitute a complex network of functions that several viruses exploit to replicate, especially RNA viruses whose cycle mainly occurs in the cytoplasm, but, unexpectedly, many viral components import in the nucleus and interact with nucleolar factors (Hiscox, 2007).

Mostly, the N protein and core proteins appear to exploit their ability to bind to the RNA molecule to interact with the nucleolar RNA and specifically localize there. This is the case for the majority of coronaviruses (Wurm et al., 2001), whose N protein probably share a so-called nucleolar retention signal sequence (Reed et al., 2006). Studies with deletion mutants and complementation assays coupled to imaging techniques suggest that the N protein may act as cytoplasm/nucleolus shuttle protein (Timani et al., 2005). The relocation of the N protein to the nucleolus seems cell cycle-dependent, with a greater accumulation in G2/M phase (Cawood et al., 2007). Not only N protein but aslo nsp3b (ORF3b) protein of SARS-CoV was found to colocalize with C23 (nucleolin) and B23 (nucleophosmin) (Yuan et al., 2005). So far, for the newly discovered SARS-CoV-2, it is still under investigation where N proteins may localize to the nucleolus. Of note, Wu et al. (2020), through a machine-learning approach, predicted the 5′ and 3′ SARS-CoV-2 genomic untranslated regions to be enriched in the host mitochondrial matrix and nucleolus (Table 1). Therefore, the localization of SARS-CoV-2 genetic material to the nucleolar body might be essential for viral life cycle, but more extensive research is demanded to clarify the interplay between SARS-CoV-2 N protein and the nucleolus.

HIV-1 has, as well, evolved mechanisms that involve the nucleolus; indeed, both Tat and Rev proteins have been characterized with a nucleolar localization signal since the end of the last century (Cochrane et al., 1990; Siomi et al., 1990). Tat constitutive expression in T cells resulted in a specific nucleolar proteomic profile with 49 proteins displaying a significant fold change compared to control. The ribosomal proteins and ribosomal biogenesis enzymes were in the top 20 most enriched ones, suggesting an HIV-induced upregulation and usurpation of ribosomal cellular machinery (Jarboui et al., 2012). On the other hand, since Rev protein is involved in intron-containing vRNA export, it was speculated that the nucleolus might be the platform for the assembly of RNP particles containing HIV-1 RNA genome and viral/cellular factors, which were then exported to the cytoplasm (Table 1; Michienzi et al., 2000). Indeed, upon Rev expression, the relocation of the nucleoporins Nup98 and Nup214 was observed, along with the export-aiding protein CRM1 in the nucleolus of HeLa cells (Zolotukhin and Felber, 1999). In particular, it was shown in live that Rev multimerizes in the nucleolus (Daelemans et al., 2004). Because of the relevance of Tat and Rev in a relatively early stage of viral replication, it has been proposed to target their activity for therapeutic purposes. Lastly, in the recent years, HIV-1 NC was also found to have a nuclear and nucleolar localization (Yu et al., 2016), but while several studies are carried on to determine its functions in the nucleus, little is known about the functional characterization of nucleolar nucleocapsid localization. Even if at first sight the nucleolus might be only a ‘ribosomal machine’, it seems that viruses have interest to interact with nucleolar proteins and accumulate viral components there.

Overall, the data accumulated so far highlight not only the importance of the impairment and reprogramming of cellular protein synthesis for viral life cycle but also the exploitation of this nuclear MLO for genomic and subgenomic storage and/or as RNP assembly platform for nuclear export, facilitating vRNA trafficking.

### PML NBs

PML NBs were identified by electron microscopy in several cell types (de Thé et al., 1960) and may vary from 0.1 µm to 1.0 µm in diameter size. These nuclear superstructures are formed by the phase separation of multiple cellular proteins, which accumulate to be SUMOylated (Bernardi and Pandolfi, 2007). The key proteins of these structures are Sp100, hDaxx, and PML. Because of the importance of SUMOylation in the regulation of a variety of cellular functions, PML NBs are involved in stress-related as well as homeostatic processes: stress response, oncogenesis, gene regulation, cell senescence, DNA damage repair, apoptosis, and antiviral response. In the context of viral infection, it has been demonstrated that PML and Sp100 expression is directly induced by IFN treatment (Everett and Chelbi-Alix, 2007). Recent studies show how PML NBs may have a role in HIV-1 persistence, since silenced HIV-1 proviruses are found in close proximity to PML in T lymphocytes (Table 1; Lusic et al., 2013). The HIV-1/PML proximity is lost upon chemical cell activation or upon inhibition of Class I HDACs. Indeed, HIV-1 activation is induced by PML knockdown and seems very specific and related to the loss of PML-associated repressive chromatin modifications, such as H3K9me2. With a view to novel therapeutic targets, the importance of oxidative stress and iron metabolism in HIV-1 infection has been highlighted and PML NBs seem to be the central players (Shytaj et al., 2020). Particularly, upon infection, PML proteins are hyper-SUMOylated and degraded with concomitant active viral expression; on the contrary, upon antioxidant treatment or iron chelation, HIV-1 reestablishes a latent phenotype and PML levels are restored (Shytaj et al., 2020).

### Nuclear speckles

Nuclear speckles (NSs) are highly versatile condensates of ~0.3–3 µm. Their characterization started with two major discoveries by Ramón and Cajal (‘El nucleo de las celulas piramidales del cerebro humano y de algunos mamiferos’, 1910) and independently, later on, by Swift (1959), who identified through electron microscopy structures that he named ‘interchromatin granule nuclear clusters’. The nowadays term was given by Beck (1961). Anatomy and function of NSs started to become clearer when these structures were re-identified through immune-labelling of some components of the pre-mRNA splicing machinery, like snoRNPs, spliceosome subunits, and other splicing factors, as well as through the highlighting of clusters of polyA^+^ RNAs (Spector et al., 1991; Carter et al., 1993). The functions of NSs are still under investigation, but they oscillate between two major processes: the storage and posttranslational modification of splicing machinery components, which is supported by the presence of several phosphatases and kinases in NSs, and the ability of being molecular hubs of transcriptional expression linking different loci to the same RNA-processing factory (Sutherland and Bickmore, 2009). Key components of NSs are the protein SC35, the scaffolding protein SON (Sharma et al., 2010), and the lncRNA MALAT-1 (Fei et al., 2017); the latter is highly enriched in NSs and has a role in the recruitment of splicing factors to nascent transcripts. The splicing is an essential step for HIV-1 replication. Indeed, some studies identify a link between speckles factors and HIV-1 infection. Two- to three-fold increase in the expression of SC35 RNA was detected upon infection (Maldarelli et al., 1998), indicating that HIV infection alters speckle factors and probably their composition. In fact, in human macrophages, SC35 protein levels were upregulated in the first weeks of infection probably favoring the splicing of vRNA, while hnRNPs, which are inhibitory factors, were downregulated. In addition, a correlation with Tat expression was observed when SC35 levels were higher (Dowling et al., 2008), supporting the role of splicing factor levels in HIV-1 replication–persistence balance.

## Pandemic viruses trigger the formation of condensates as the scaffold for their replication

**Figure 1:**
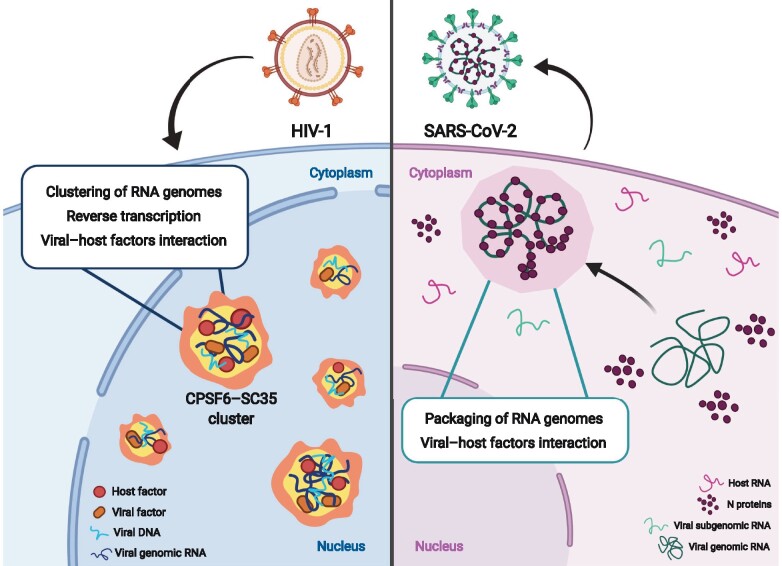
MLOs induced by HIV-1 and SARS-CoV-2 to replicate. Left: HIV-1 infection prompts the formation of nuclear MLOs enriched with host factors, such as CPSF6 and SC35, and in viral components, such as vRNA, vDNA, capsid, and integrase. Right: SARS-CoV-2 N protein forms condensates in the cytoplasm to recruit exclusively intact vRNA genome against subgenomic vRNAs or host RNAs. Cartoon created with BioRender.com.

Condensation and coalescence of host/viral components can create a favorable environment for viral replication. This microenvironment is formed by MLOs created from LLPS enhanced by viral infection. Recently, it has been surprisingly found that HIV-1 infection remodels the nuclear intracompartments. In particular, HIV-1 relocates and condensates a paraspeckle factor, CPSF6, in a different MLO enriched in speckle factors, such as SC35 (Francis et al., 2020; Rensen et al., 2020; Scoca et al., 2020). This viral action most probably turns the host speckles in new hubs for viral replication. Despite the fact that the HIV was discovered in 1983 (Barre-Sinoussi et al., 1983), only recently, new insights into reverse transcription were tracked down. The reverse transcriptase (RT) enzyme was at the basis of the HIV discovery, thanks to the detection of RT activity in the supernatant of cellular culture, which permitted to understand the nature of the isolated retrovirus, directly from a lymph node biopsy of an HIV-infected patient. HIV-1 relies on the retro-transfer of RNA genetic information to DNA to be able to integrate into the host genome to ensure viral persistence. Notably, the ‘central dogma’ based on the concept that the genetic information could be carried only by the DNA was revised. Historically, the discovery of RT (Baltimore, 1970; Temin and Mizutani, 1970) has been considered a milestone of molecular biology and biotechnology, enabling scientists to set up new tools that heavily influenced cloning, analysis of gene expression, the study of RNA biology, and the development of state-of-the-art technologies and the modern medicine. Of note, the RT discovery has been recompensed with the Nobel Prize to three scientists: Renato Dulbecco, Howard Temin, and David Baltimore. HIV RT has been the most exploited antiviral drug target ever and, to date, 12 anti-RT drugs have been developed: nucleoside RT inhibitors and nonnucleoside RT inhibitors. So far, the RT activity has been considered a process that begins and ends in the cytoplasmic compartment of the host cell. However, a recent study directly highlights the presence of a nuclear RT activity in the nucleus of infected macrophages, revisiting the HIV RT dogma considering that retrotranscription can occur exclusively in the cytoplasm of the host infected cell (Rensen et al., 2020). Of note, in the HIV-1-induced CPSF6–SC35 MLOs, newly synthesized viral DNA (vDNA) was found (Rensen et al., 2020; Scoca et al., 2020). These results point out that, at least in macrophages, the nuclear reverse transcription can occur, likely ending inside the nucleus, contrary to the current belief. Other recent studies (Burdick et al., 2020; Dharan et al., 2020; Selyutina et al., 2020) supported similar conclusions on the spatiotemporal action of RT. This surprising discovery of the new cellular compartment where retrotranscription can occur has been elucidated only after 37 years from the HIV discovery (Barre-Sinoussi et al., 1983) and after 50 years from the stunning finding of the RNA-dependent DNA polymerase RT (Baltimore, 1970; Temin and Mizutani, 1970). Interestingly, the HIV genomic RNA has been observed in the host nuclei from several groups, suggesting that RT completion is dispensable for nuclear entry (Burdick et al., 2013, 2017, 2020; Bejarano et al., 2019; Dharan et al., 2020; Selyutina et al., 2020). However, the physiological role of the presence of RNA inside the nucleus of infected cells was obscure. The discovery of the formation of RNA genome clusters englobed in nuclear MLOs gave the opportunity to visualize for the first time the presence of a nuclear RT activity using cutting-edge imaging technologies (Table 1; Rensen et al., 2020). HIV genomes cluster together with host factors, such as CPSF6 and SC35, usually located in distinct nuclear locations, paraspeckles and speckles, respectively. This finding supports the hypothesis that HIV generates novel MLOs for its own aim or induced by the host to control the fuel of infection (Figure 1). Several speculations can be proposed to explain the presence of these viral/host nuclear structures: (i) to serve as nuclear microreactors that condensate RT enzymes and host factors to promote vDNA synthesis in macrophages; (ii) to serve as microenvironments that include viral and host factors required for the generation of new viral progeny and/or to hide the virus from cellular defense mechanisms (Schmid et al., 2014; Lahaye et al., 2018); (iii) related to the finding that large amounts of unintegrated vDNA cluster in the nucleus, probably forming viral reservoirs, which constitute the bottleneck for a cure against HIV; and (iv) HIV-induced MLOs located in the nucleus could serve as source of storage of viral genomes ready to be packed to spread as new progeny. HIV genome clusters co-localize with condensates formed by SC35, a known speckle factor (Francis et al., 2020; Rensen et al., 2020). Whether or not speckles have a role in HIV integration and transcription still should be clarified, because current technologies used to visualize HIV DNA, such as click chemistry based on EdU incorporation during the ongoing RT, interfere with viral transcription and are not sufficiently sensitive to visualize an individual viral genome (Rensen et al., 2020). However, this technology can successfully label nascent HIV DNA in nondividing cells. In fact, episomal forms of HIV DNA containing EdU have been visualized in MLOs enriched with CPSF6 and SC35 (Rensen et al., 2020). On the other hand, more powerful imaging technologies are needed to identify the location of viral proviruses, in particular by using live imaging. HIV-1 ANCHOR technology offers good potentiality to investigate this important step of viral life cycle, because it allows the visualization of single PIC (Blanco-Rodriguez et al., 2020; Scoca et al., 2020) using live imaging. Recent results show that HIV-1 infection induced remodeling of preexisting MLOs generating HIV-1 MLOs, thus it is possible that the remodeling of the nucleus by HIV-1 (Table 1; Scoca et al., 2020) regulates viral transcription. Future studies will aim to define whether speckles or HIV-induced nuclear MLOs are involved in fueling viremia or in the persistence process promoted by HIV to ensure its own survival in the host. In addition, LLPS contributes to the formation of heterochromatin and nucleosomes, which have the intrinsic property to phase-separate from the rest of the nuclear environment. The phase-separating properties of several chromatin-binding proteins seem to be essential for the regulation of chromatin dynamics and transcription (Hnisz et al., 2017; Larson et al., 2017; Strom et al., 2017; Boehning et al., 2018; Boija et al., 2018; Cho et al., 2018; Sabari et al., 2018; Nair et al., 2019; Plys et al., 2019; Zhang et al., 2019). For example, the transcriptional repressor HP1 mediates the formation of heterochromatin by its ability of phase-separating (Larson et al., 2017; Strom et al., 2017) as well as CBX2 subunit of Polycomb-repressive complex 1 can phase-separate *in vitro* (Plys et al., 2019). In contrast, other chromatin factor, such as the transcriptional coactivator BRD4, a well-known marker of euchromatin, is able to induce the formation of liquid-like condensates at super-enhancer regions (Sabari et al., 2018). Importantly, it has been reported that HIV-1 recurrently targeted host genes (RIGs) are proximal to super-enhancers genomic elements with BRD4 signature. Those RIGs cluster in particular spatial locations during the activation of T cells (Lucic et al., 2019). Whether euchomatin- or heterochromatin-related nuclear factors can establish condensates that may play a role in HIV-1 gene expression is a fascinating new perspective. The interplay between MLOs and HIV-1 genome puts forward new frontiers for future research. New results in this field could unveil mechanisms related to HIV-1 MLO function and the remodeling to regulate viral latency and viral rebound.

Similar to HIV-1, SARS-CoV-2 seems to use LLPS to spread, but in a different cellular compartment. It has been reported that N protein forms condensates in the presence of SARS-CoV-2 genome. In particular, there are several evidences suggesting that the SR-rich sequence of N protein serves as a key regulatory hub. Likely, N protein is linked to the function of the replication transcription complex (RTC). It has been observed that at early time post-infection, SR regions of N protein are phosphorylated at multiple sites by cytoplasmic kinases (Cong et al., 2020). The phosphorylated N protein associates with the RNA helicase DDX1, which induces RNA structural changes required for transcription of subgenomic RNAs (Wu et al., 2014; Carlson et al., 2020). Interestingly, a liquid-like matrix composed of phosphorylated N protein, linked to RTC membranes by Nsp3, creates a compartment to concentrate and protect the viral replication and transcription machinery. Similar mechanisms have been highlighted for negative-sense RNA viruses, where replication depends on dynamic biomolecular condensates (Table 1; Iserman et al., 2020; Perdikari et al., 2020; Savastano et al., 2020; Cubuk et al., 2021). Of note, it has been elegantly shown by the group of Gladfelter that genomic RNA carrying 5′ and 3′ ends promotes condensates in the presence of nucleocapsid (Iserman et al., 2020). Contrariwise, host RNA and viral subgenomic RNA are excluded from LLPS (Iserman et al., 2020; Figure 1). This is an appealing model for viral replication based on liquid-like droplets as hubs of viral progeny. Future studies will clarify whether this exciting model can be validated in the context of a real infection with SARS-CoV-2.

## Conclusions

A role of LLPS in pandemic viruses, such as HIV-1 and SARS-CoV-2, has been highlighted by several studies. In particular, the establishments of new condensates during viral infection formed by viral factors with or without the host factor contribution seem to be key for viral replication. Interestingly, viruses evolved independent strategies to replicate by forming condensates in the host cells. The N protein of SARS-CoV-2 condensates in the cytoplasm with the viral genome to favor viral assembly in the cytoplasmic compartment (Figure 1). The evolving research on SARS-CoV-2 replication cycle will address important mechanisms of establishment of viral MLOs. In contrast to SARS-CoV-2, HIV-1 replicates in the nuclear compartment inducing new MLOs (Figure 1), containing HIV-1 RNA genome, capsid, and integrase from the incoming viral particles together with host nuclear factors. Several important functions have been attributed to these viral/host nuclear organelles, such as HIV-1 RNA genome sequestration, RT activity, and interaction with host factors, such as splicing components (Figure 1). These HIV-induced MLOs locate in SC35-speckle regions to reprogram preexisting organelles to obey to the viral needs. Thus, viral MLOs could represent a new frontier of therapeutic targets to block viral replication either at early or late stages of viral life cycle.

## Funding

Work in the authors’ laboratories was supported by the Agence Nationale de Recherche sur le SIDA (ANRS)/REACTing grant ECTZ88162 with a nominative PhD student fellowship ECTZ88177 for V.S. and by the Pasteur Institute.

**Conflict of interest:** none declared.
